# Supplementary material for: Community awareness, perceptions, and management practices related to pre-eclampsia: An exploratory qualitative study in Mbale City, Eastern Uganda
Source: PLoS One. 2026 Mar 4;21(3):e0314674. doi: 10.1371/journal.pone.0314674 (PMC12959696; doi:10.1371/journal.pone.0314674)
Supplement: Attachment — Submitted filename: Rebbuttal- Plos One vr 10.pdf [file pone.0314674.s007.pdf]

25/May/2025
Enid Kawala Kagoya
Busitema University
Faculty of Health Sciences
enidkawala@gmail.com

To: The Editors
PLOS ONE

Subject: Resubmission of Revised Manuscript : Women's Awareness, Perceptions of Susceptibility, Risk Factors, Responses, and Preventive Measures for Pre-eclampsia : An Exploratory Qualitative Study in Mbale City, Eastern Uganda

Dear Editors,

I am writing to submit the revised version of our manuscript entitled "Exploring Women's Awareness and Perceptions of Pre-eclampsia in Mbale City, Uganda: An Exploratory Qualitative Study" (originally titled *"Community Perceptions, Knowledge, and Experiences of Pre-eclampsia: An Exploratory Study in Uganda"*), in response to the valuable comments and guidance provided by the reviewers and editorial team.

We sincerely thank the reviewers for their constructive feedback, which has helped us significantly improve the clarity, consistency, and rigor of our manuscript. Below, we provide a point-by-point response to each comment, highlighting the specific revisions made.

Reviewer Comments and Our Responses

1. Topic: I suggest this variable be replaced with awareness.
Response: We agree with the suggestion. The word *"knowledge"* has been replaced with *"awareness"* throughout the manuscript, including in the title, abstract, objectives, results, and discussion sections to reflect the study's focus more accurately.

2. Introduction: Is it middle-income countries, kindly check and correct.
Response: Thank you for pointing this out. We have revised the phrase to "low- and middle-

income countries (LMICs)” to accurately reflect the terminology and corrected it in the Introduction section.

3. Abstract: Knowledge – check my comment on the topic suggesting to replace knowledge with awareness.
Response: The abstract has been revised accordingly. All references to *knowledge* have been replaced with *awareness* to align with the revised study focus.

4. Methodology: Was this an individual face-to-face or focus group discussion? How were they sampled?
Response: We clarified in the methodology that this was an individual face-to-face interview study using purposive sampling. These details have been added in the "Data Collection" and "Sampling" subsections.

5. Introduction: How many studies, where were they conducted? Only one reference was used for the stated sentence on studies.
Response: We have reviewed and updated the introduction to include multiple references supporting the statement. The revised sentence now cites studies from Uganda, Kenya, and Nigeria to provide a more comprehensive background

6. Objective: Knowledge – check my comment on the topic suggesting to replace knowledge with awareness.
Response: Corrected. The objective now reads: *“To explore women's awareness, perceptions of susceptibility and risk factors, community responses, and understanding of preventive measures related to pre-eclampsia in Mbale City, Uganda.*

7. Methodology: What was the reason for using this study design , is rationale.
Response: We added a rationale for the use of a qualitative exploratory design. The revision explains that it was chosen to gain in-depth understanding of women's lived experiences and contextual perceptions, which are best captured through qualitative inquiry.

8. Study area: Was this the reason why the study was conducted there? Link the study setting to the problem under study.

Response: The study area description has been revised to explicitly link Mbale City's maternal health challenges and limited community awareness of hypertensive disorders to the rationale for selecting this location.

9. Study population: What was the rationale for using these women? The study says community, not women.
Response: We have clarified that although the study refers to community-level perspectives, the focus was on women of reproductive age, as they are most affected by pre-eclampsia. The rationale for their inclusion has been added in the Study Population section.

10. Data collection: How many participants were interviewed? Was it face-to-face? How many were involved in data collection? Is it possible to interview 81 participants in 2 days? Duration of interviews?
Response:

- 81 women participated in individual face-to-face interviews.
- 28 trained research midwives were involved in data collection.
- The data collection took place over 5 days, not 2 as previously stated (corrected).
- Each interview lasted approximately 30–45 minutes.
  All of this has been clarified in the "Data Collection" section.

11. Results section: Suggest reorganizing results using themes and subthemes. Some results not in the table. Management and prevention of pre-eclampsia not in the topic. Provide at least 3 quotes per subtheme.
Response: The Results section has been completely reorganized to reflect clearly defined themes and subthemes.

- Figure 1 has been updated to reflect the revised structure.
- At least three illustrative quotes have been included per subtheme.
- Sections unrelated to the main topic (e.g., management) have been renamed or reframed to align with *awareness and perceptions*.

12. Above are 4 themes under analysis, kindly correct.
Response: Corrected. The analysis framework now clearly outlines five major themes in both text and visual representation (Figure 1).

13. Replace knowledge with awareness.
Response: Done throughout the manuscript.

14. Subtheme one: Knowledge of susceptibility and risk factors.
Response: Revised to “Awareness of susceptibility and risk factors” for consistency.

15. Theme 3: Management of pre-eclampsia – Not part of the topic; restructure.
Response: This theme has been reframed as “Awareness of treatment options for pre-eclampsia”, maintaining alignment with the study focus.

16. Subtheme: Community response to pre-eclampsia – This can work as a theme.
Response: Accepted. This has been elevated to a standalone theme in the Results section.

17. Theme 4: Prevention of pre-eclampsia – restructure as awareness of preventive measures.
Response: Revised to “Awareness of preventive measures for pre-eclampsia” and structured accordingly.

18. Discussion: Correct the results section, and this also to be corrected.
Response: The Discussion has been updated to reflect the restructured Results section, with aligned interpretation and reflection on the revised themes.

19. Conclusion: Restructure based on findings.
Response: The Conclusion has been rewritten to accurately reflect the study findings and their implications for public health education and maternal care services.

20. Inside citation are 14, in section, is only 10. Correct and include all resources.
Response: All citations have been reviewed. The reference list has been updated to include all cited sources. In-text citations have been cross-checked for completeness and accuracy.

We trust that these revisions address all the concerns raised by the reviewers. Thank you for your time and the opportunity to revise and resubmit our manuscript.

Yours sincerely,

Enid Kawala Kagoya
Corresponding Author
Busitema University, Mbale, Uganda

1. On the topic:I suggest this variable be replaced with awareness
2. Introduction: Is it middle income countries, kindly check and correct
3. Abstract: Knowledge , check my comment on the topic suggesting to replace knowledge with awareness
4. On methodology: Was this an individual face to face or focus group discussion, how were hey sampled
5. Introduction: How many studies, where were they conducted, check, only one reference was used for the stated sentence on studies
6. Objective: Knowledge , check my comment on the topic suggesting to replace knowledge with awareness
7. Methodology: What was the reason for using this study design, is rationale
8. Study area: Was this the reason why the study was conducted there, I suggest the authors link the study setting to the problem under study
9. Study population: What was the rationale of using these women, the study says community not women, kindly check and correct
10. Data collection: How many participants were interviewed, was this face to face interviews or what. How many people were involved in data collection, I have seen about 81

participants, is this possible to interview 80 patients in 2days, how many minutes did each interview last

11. Results section: Iam lost when I read this section, I suggest the authors re look at their results and structure them starting from the presentation as themes and subthemes, let figure 1 clearly present the results suggested. When reading through this section, there are results which are presented which are not in the table, the management and prevention of preeclampsia os not part on the topic. Kindly relook at this section, when presenting your results, kindly start by the main theme, talk to it then each subtheme to be presented supported by relevant quotes atleast three quotes per subtheme will be sufficient
12. Above is 5 themes under analysis, kindly correct
13. Replace knowledge with awareness
14. Subtheme one: knowledge of susceptibility and risk factors
15. Theme 3: management of preeclampsia: Not part of the topic; make sure you can restructure the naming of the theme to suite the topic
16. Subtheme: Community response to preeclampsia: This one can work as theme
17. Theme 4: Prevention of preeclampsia: not part of the topic, may be can be structured as awareness of preventive measures of preeclampsia
18. On discusiion: Correct the results section and this also to be corrected
19. Conclusion: Restructure basing on the conclusion from the findings
20. Inside citation are 14, in section, is only 10, kindly correct and include all resources

We have carefully considered all feedback and made thorough revisions to improve the clarity, depth, and scientific rigor of the manuscript. Specifically, we have addressed each reviewer’s comment point by point, revised key sections including the title, Abstract, introduction, methods, results, and discussion, and enhanced alignment between the study objectives, findings, and conclusions.

We are grateful for the constructive feedback, which has significantly strengthened our work. We hope that the revised manuscript meets the expectations of the journal and look forward to your favorable consideration.

Thank you for your time and continued guidance.

Sincerely,

Enid Kawala Kagoya
On behalf of all co-authors
Busitema University, Faculty of Health Sciences.
