# Supplementary material for: Community awareness, perceptions, and management practices related to pre-eclampsia: An exploratory qualitative study in Mbale City, Eastern Uganda
Source: PLoS One. 2026 Mar 4;21(3):e0314674. doi: 10.1371/journal.pone.0314674 (PMC12959696; doi:10.1371/journal.pone.0314674)
Supplement: Attachment — Submitted filename: Point by point response - PLOS ONE 14.pdf [file pone.0314674.s010.pdf]

enidkawala@gmail.com

Busitema University

Faculty of Health Sciences

P.O Box 1460, Mbale, Uganda

26th/January/2026

To the Editor

PLOS ONE

Dear Sir

Ref: PONE-D-24-50330R3: Women's Awareness, Perceptions of Susceptibility, Risk Factors, Responses, and Preventive Measures for Pre-eclampsia: An Exploratory Qualitative Study in Mbale City, Eastern Uganda

We sincerely thank the reviewers for their detailed and constructive feedback. Each comment has been carefully addressed, and revisions have been made to strengthen the manuscript. Below is our point-by-point response.

First Reviewer Comment: Title and Objective Alignment

Comment: There is an inconsistency between the manuscript title and the stated objective. The title alternates between "community perceptions" and "women's perceptions," while the objective clearly identifies women of reproductive age. The title and objective should be aligned.

Response: We acknowledge this inconsistency. The title has been revised as *Community Awareness, Perceptions, and Management Practices Related to Pre-Eclampsia* to reflect the broader community awareness context. To ensure alignment, the objective has been revised to clarify that women of reproductive age within the community were the primary respondents, representing community-level awareness.

Revised Objective: To explore community awareness, perceptions, and management practices related to pre-eclampsia in Mbale City, Eastern Uganda, with a particular focus on women of reproductive age, and to examine how these factors influence health-seeking behaviors.

Methods Clarification Added: Although the study focuses on community awareness, data were collected from women of reproductive age as the primary respondents.

First Reviewer Comment 2: Study Design

Comment: The study design should be clearly described.

Response: We have clarified that an exploratory qualitative study design using face-to-face in-depth interviews was employed to capture detailed perceptions, awareness, and management practices related to pre-eclampsia in a low-resource urban setting.

First Reviewer Comment 3: Study Site

Comment: Provide more contextual information about the study site.

Response: We have expanded the Study Site section:

1. The study was conducted in Mbale City, Eastern Uganda, a newly approved city with a high burden of pre-eclampsia.
2. Health records indicate that pre-eclampsia accounted for a substantial proportion of maternal admissions and deaths in 2020-2021, highlighting the relevance of this site for research.

First Reviewer Comment 4: Study Population and Recruitment

Comment: Clarify how participants were identified and recruited, and how representativeness was ensured.

Response: Purposive sampling was employed. Trained Research Midwives approached women in markets, health facilities, community centers, streets, and selected households. Eligibility criteria: women aged 18-49 years and willing to participate.

Recruitment across multiple locations ensured diverse perspectives and reduced selection bias.

Parity Consideration: Parity was not used for stratification, but prior pregnancy experience was collected during interviews and considered in data interpretation.

Data Collection and Independence:

1. Interviews were individual and private.
2. Conducted in homes, clinics, or quiet community spaces, lasting 30=45 minutes in English. Kindly note that those working in markets allowed us to take them to safe and quite places just above the market which we did.

Second Reviewer Comment 5: Data Management and Analysis

Comment: Clarify how data were managed and analysed.

Response: Audio-recorded interviews were transcribed verbatim.

1. Transcripts were checked against recordings for accuracy.
2. Data were analyzed using ATLAS.ti with thematic analysis.
3. Five main themes and subthemes emerged.

Re-analysis for Data Saturation (New):
In response to the reviewer's suggestion regarding the limited use of quotes and potential for multiple papers:

- We conducted a thorough re-analysis of all transcripts.
- Additional codes were extracted and mapped under each theme and subtheme to capture the richness of the dataset.
- For each subtheme, we now include at least three representative quotes, reflecting diverse participants, experiences, and perspectives.

Explicit references to Table 1 were added throughout the Results section to link narrative descriptions to the structured thematic summary.

- These revisions demonstrate data saturation and substantiate the findings more robustly.

Second Reviewer Comment: Subthemes Accuracy

Comment: Check correctness of subthemes such as balanced diet, ANC attendance, and exercise.

Response: We reviewed these subthemes and confirm they accurately reflect participant narratives.

1. No additions or deletions were necessary, and these have been retained.

Second Reviewer Comment 7: Potential for Multiple Papers

Comment: The dataset is rich and could support two to three separate publications.

Response:

✓ We acknowledge the richness of the dataset.

- ✓ For this manuscript, we focused on the primary study objective, presenting comprehensive themes and subthemes.
- ✓ The re-analysis and inclusion of additional codes and quotes strengthens this manuscript's contribution.
- ✓ Future analyses could explore cultural beliefs, management practices, or preventive strategies as separate manuscripts.

Thank you for the opportunity to revise our manuscript. We appreciate the reviewer's constructive feedback and have carefully addressed all the suggested minor revisions to improve the clarity and presentation of the study.

Warm regards,

Dr. Enid Kagoya Kawala
Corresponding Author
Busitema university
enidkawala@gmail.com
