# Supplementary material for: Community awareness, perceptions, and management practices related to pre-eclampsia: An exploratory qualitative study in Mbale City, Eastern Uganda
Source: PLoS One. 2026 Mar 4;21(3):e0314674. doi: 10.1371/journal.pone.0314674 (PMC12959696; doi:10.1371/journal.pone.0314674)
Supplement: Attachment — Submitted filename: POINT BY POINT RESPONSE - Plos One.docx [file pone.0314674.s005.docx]

**POINT-BY-POINT RESPONSE**

**PLOS One**

**20/03/2025**

| **No** | **Comment** | **Response** | **Liner number (s) in tracked changes** | **Page number (s) in tracked changes** | **Liner number (s) in clean copy** | **Page number (s) in clean copy** |
| --- | --- | --- | --- | --- | --- | --- |
| 1 | The abstract is well written, however, under methods, clarify if the interviews conducted were individual face-to-face or focus group, and also add how the 81 participants were sampled. | Thank you so much for this observation,  All interviews were face-to-face interviews with the mothers as indicated in the review. | 59 | 3 | 59 | 3 |
| 2 | On the background, there was a mention of the high level of studies that were conducted, kindly clarify how many studies, where were they conducted, and recheck as only one reference was used for the stated sentence on studies.  . | Thank you so much for this keen observation, this has been rectified | 103 | 4 | 103 | 4 |
| 3 | Under study design, clarify the reasons for using qualitative research design, and your rationale. | Thank you so much for this comment  The rationale for using a qualitative study design was to gain an in-depth understanding of the community's knowledge, perceptions, and experiences related to pre-eclampsia. Qualitative methods were ideal for exploring the complex and subjective nature of health beliefs, cultural practices, and personal experiences. This approach allowed the researchers to uncover local perspectives on pre-eclampsia, identify misconceptions, and understand the factors influencing health-seeking behavior. By using in-depth interviews, the study captured the cultural context and social determinants that shaped women's knowledge and decisions regarding care, providing valuable insights for designing targeted educational interventions | N/A | N/A | N/A | N/A |
| 4 | Under the study setting, agriculture was mentioned and linked to the study setting, was this the reason why this study was conducted there? I suggest the authors link the study setting with the problem under study, for example, having a higher number of women with pre-eclampsia or hypertensive disorders in pregnancy. The accessibility and utilisation rate of the health care facility, the number of pregnant women seen daily, the number of midwives to cater for these women. The number of hospitals or health care facilities that are catering for pregnant women etc | Thank you for the observation, this has been rectified | 116-119 | 4 | 116-119 | 4 |
| 5 | Under the study population, 18-49 women were the population, what was the rationale of using these women, the title says community not women, kindly recheck and correct this. | Thank you for this observation. This has been rectified as below  The study population consisted of women aged 18-49 years from the community of Mbale City, Uganda, who were recruited during a community outreach initiative on pre-eclampsia | 121-122 | 5 | 121-122 | 5 |
| 6 | Before data collection, how were the participants sampled, and the rationale of the sampling method, kindly add a sub-heading on sampling. | Thank you so much for this comment, this section has been added | 124-130 | 5 | 124-130 | 5 |
| 7 | . Under data collection, line number three indicated that data was collected for the period of 2 days, how many participants were interviewed, was these face-to-face interviews or what, how many people were involved in data collection? I have seen about 81 participants, is it possible to interview 81 women in two days? How many minutes did the interviews last with each participant, where was it conducted, more information is needed. What guided the authors to say they have gathered enough data? Which language was used during data collection? | Thank you so much for this observation  Data collection was conducted over a period **of six days** (from 21st to 26th May 2024), not two days as mentioned. During this period**, 81 women** aged 18-49 years were interviewed. The data collection involved **face-to-face in-depth interviews**, which allowed for detailed exploration of the participants' knowledge, perceptions, and experiences related to pre-eclampsia. A team of researchers and trained field assistants conducted the interviews, ensuring consistency and reliability in data gathering.  Each interview lasted approximately **30 to 45 minutes**, providing sufficient time to delve into the participants' understanding and personal experiences regarding pre-eclampsia. Interviews were conducted in **Mbale City, Uganda**, in community-based settings such as local health centers, community halls, and participants' homes to ensure accessibility and comfort for the women being interviewed.  The researchers determined that data saturation had been reached when no new themes or significant information emerged from the interviews, suggesting that enough data had been gathered to address the research objectives comprehensively. The **interviews were conducted in english** to ensure clarity and facilitate open communication however tis has been rectified | 132-144 | 5 | 132-144 | 5 |
| 8 | Under the results, I was lost when I was reading this section, I suggest the authors to re-look at their results and structure them starting from the presentation as themes and sub-themes. Let figure 1 present the results as suggested (themes and sub-themes). When reading through this section, there are results which are presented which are not in the figure. The sub-theme on management and prevention of pre-eclampsia is not part of the topic. Kindly re-look at this section. When presenting your results, kindly start with the main theme, talk to it, then each sub-theme to be presented, supported by relevant quotes at least three quotes per sub-theme will be sufficient. Address this section, after addressing it, kindly recheck your discussion to align with the corrections suggested. Check my other comments in the paper. Align your conclusion with the study findings. | Thank you so much and apologies for that  Kindly take a look at the revised results section. | 167-504 | 6-20 | 167-504 | 6-20 |
| 9 | References, inside citations, are 14, in this section are only ten, kindly correct and include all the sources | Thank you so much, I have included all of them | 597-640 | 23-25 | 597-640 | 23-25 |
