# Supplementary material for: Community awareness, perceptions, and management practices related to pre-eclampsia: An exploratory qualitative study in Mbale City, Eastern Uganda
Source: PLoS One. 2026 Mar 4;21(3):e0314674. doi: 10.1371/journal.pone.0314674 (PMC12959696; doi:10.1371/journal.pone.0314674)
Supplement: Attachment — Submitted filename: Point by point response - PLOS One Vr 12.docx [file pone.0314674.s009.docx]

**Response to Editorial Requests – PONE-D-24-50330R3**

**Manuscript Title:** Women's Awareness, Perceptions of Susceptibility, Risk Factors, Responses, and Preventive Measures for Pre-eclampsia: An Exploratory Qualitative Study in Mbale City, Eastern Uganda

**Author:** Ms. Enid Kagoya Kawala

**Dear PLOS ONE Editorial Team,**

Thank you for your message and for the opportunity to revise and resubmit our manuscript. Please find below our detailed, point-by-point responses to the editorial queries.

**Summary Table of Responses**

| **Editorial Comment** | **Author’s Response / Action Taken** |
| --- | --- |
| **1. Multiple versions of submission files**   “We note that there were multiple versions of 'Manuscript, Cover Letter, Revised Manuscript with Track Changes and Response to Reviewers' in your submission's file inventory. We have removed the older files, retaining the most recent versions for editorial review. Please double check your submission file inventory and let us know if any files appear to be outdated or absent.” | We have reviewed all files under the submission inventory in Editorial Manager. The following files are now retained as the most recent and accurate versions:  • Clean revised manuscript (Vr 12)  • Manuscript with tracked changes vr 12 • Response to reviewers document  • Updated cover letter  All outdated files have been deleted to avoid confusion. We confirm that the retained files represent the most up-to-date versions for editorial review. |
| **2. Missing Figure 1**   “Please upload a copy of Figure 1 which you refer to in your text on page 6. Or if the figure is no longer to be included as part of the submission please remove all reference to it within the text.” | Figure 1 has now been changed to table 1. |
