# Supplementary material for: Community awareness, perceptions, and management practices related to pre-eclampsia: An exploratory qualitative study in Mbale City, Eastern Uganda
Source: PLoS One. 2026 Mar 4;21(3):e0314674. doi: 10.1371/journal.pone.0314674 (PMC12959696; doi:10.1371/journal.pone.0314674)
Supplement: Attachment — Submitted filename: Reviewer.docx [file pone.0314674.s008.docx]

**Reviewer’s Comments**

Here, I've forwarded the feedback below. Please make the necessary changes to the manuscript in response to the comments.

**Title:**

There appears to be a discrepancy between the title and the stated objective of the manuscript. The title refers at some points to community perception, while in other places it refers to women’s perception.

In addition, the objective specifies women of reproductive age as the study population. This inconsistency makes it unclear who the actual study population is. I recommend that the authors revise the title and objective to ensure alignment and clarity regarding whether the focus is on the community at large or specifically on women of reproductive age.

**Method**:

 Recruitment **and Access to Participants**

- The manuscript states that participants were recruited during a community outreach initiative. However, it is not clear how women of reproductive age were specifically identified and approached within this outreach?
- More detail is needed on the recruitment strategy to ensure representativeness and minimize selection bias.
- The study population includes women aged 18–49 years, but the methods do not indicate whether parity (primigravida vs. multiparous) was considered during recruitment or analysis. Since knowledge and perceptions of pre-eclampsia may differ significantly between first-time mothers and those with prior pregnancies?
- The authors should clarify the data collection approach. Specifically, were interviews conducted through house-to-house visits with eligible women, or were participants interviewed collectively at a single venue during the community outreach program? And how independence of responses was ensured?

**RESULT**:

While many participants incorrectly believed that pre-eclampsia was related to high blood pressure resulting from stress; is this in your result misunderstanding?

**Clarity of Misconceptions**

- The authors should clarify whether the reported misconceptions about pre-eclampsia were common among most participants or only mentioned by a few. It is important to indicate how many participants held misconceptions versus those with accurate understanding?
